# From Invisible to Visible: Cutting-Edge Ultrasound Insights into Entheses of the Distal Extremities in Rheumatology

**DOI:** 10.3390/jcm15103753

**Published:** 2026-05-13

**Authors:** Luis Coronel, Chiara Rizzo, Juan José de Agustin, David Bong, Maribel Miguel-Pérez, Stefano Alivernini, Lene Terslev, Maria Antonietta D’Agostino, Ingrid Möller

**Affiliations:** 1 Rheumatology Unit, Image and Techniques Section, Vall d´Hebron Hospital Universitari, Vall d´Hebron Barcelona Campus, 08035 Barcelona, Spain; 2 Instituto Poal de Reumatologia, 08022 Barcelona, Spain; 3 Unit of Human Anatomy and Embryology, Department of Pathology and Experimental Therapeutics (Campus of Bellvitge), University of Barcelona, 08907 Barcelona, Spain; 4 Division of Rheumatology and Clinical Immunology, Fondazione Policlinico Universitario Agostino Gemelli IRCCS, 00168 Rome, Italy; 5 Division of Rheumatology, Università Cattolica del Sacro Cuore, 00168 Rome, Italy; 6 Copenhagen Center for Arthritis Research, Center for Rheumatology and Spine Diseases, Rigshospitalet, 2600 Glostrup, Denmark; 7 Department of Clinical Medicine, University of Copenhagen, 2200 Copenhagen, Denmark

**Keywords:** enthesis, spondyloarthritis, ultrasound, biopsy, anatomy

## Abstract

Musculoskeletal ultrasound (MSUS) is a well-established and reliable tool for the evaluation of entheses and enthesitis, particularly at larger and accessible sites. Recent technological advances, including high- and ultra-high-frequency transducers, have expanded its potential, enabling detailed assessment of distal extremity entheses. This narrative review provides a focused and updated perspective on this evolving field, highlighting three key advances. First, the identification and characterization of previously underrecognized entheseal sites in the distal extremities, such as pulley systems, retinacula, novel tendon insertions, and collateral ligaments, broadening the morphological spectrum of entheseal imaging. This is complemented by improved evaluation of vascularization through microvascular imaging and contrast-enhanced US (CEUS). Second, the emergence of interventional approaches, particularly US-guided entheseal biopsy, offers a novel means to investigate entheseal tissue in vivo and may establish a link between imaging and histopathology. Third, the integration of advanced functional imaging modalities, including elastography and multispectral optoacoustic tomography (MSOT), provides preliminary additional insights into the biomechanical and molecular properties of the enthesis beyond conventional structural assessment. Collectively, these developments support new investigational perspectives, positioning MSUS as a dynamic and integrative modality capable of exploring new anatomical territories and biological dimensions, with the potential to reshape the understanding and evaluation of entheseal involvement in rheumatology.

## 1. Introduction

The enthesis is traditionally defined as the insertion site of tendons, ligaments, pulleys, fasciae, or joint capsules onto bone [1]. Biomechanically, the enthesis is a stress-concentration zone in which opposing elastic moduli are balanced to dissipate tensile forces across the transition from soft to mineralized tissue, protecting the osteotendinous junction [2]. Two main types are described: (i) fibrous entheses, which attach directly to bone or indirectly via the periosteum and are typically associated with large, powerful muscle groups (i.e., deltoid insertion at the deltoid tuberosity of the humerus); and (ii) fibrocartilaginous entheses, which insert onto more localized areas of bone and are particularly adapted to resist compressive and shear forces (i.e., where tendons wrap around the bone, such as peroneal tendons, or the Achilles tendon insertion at the calcaneus during multiaxial loading). Fibrocartilaginous entheses display a four-zone graded architecture that enables a gradual mechanical and compositional transition from tendon to bone. The first zone consists of dense fibrous connective tissue rich in type I collagen. This is followed by uncalcified fibrocartilage, containing predominantly type II and III collagen. The third zone consist of calcified fibrocartilage, which anchors the tendon to bone through interdigitation of collagen fibres with the mineralized matrix. Finally, the fourth zone corresponds to the bony cortex. The boundary between uncalcified and calcified fibrocartilage is marked by the tidemark, a basophilic front that delineates soft from hard tissue. This four-zone arrangement allows efficient force transmission, firm anchorage, and resistance to multidirectional loading during joint motion [3].

The clinical relevance of the enthesis became particularly evident in the context of the spondyloarthritis (SpA) group [4]. The concept of SpA as a distinct entity emerged in 1958, when it was recognized that certain inflammatory rheumatic diseases shared features clearly differentiating them from rheumatoid arthritis (RA) [5]. In 1970, entheseal involvement was first described as a key element in the pathogenesis of these conditions [6]. Subsequent work established enthesitis as a central lesion underlying many of their clinical manifestations. At the axial level, inflammation at spinal and sacroiliac entheses contributes to inflammatory back pain, chest wall pain, stiffness, and functional limitation. Peripherally, the involvement of entheses is a pivotal feature of psoriatic arthritis (PsA). It explains common features such as Achilles, patellar or common extensor tendon (CET) inflammation and plantar fasciitis, and is also implicated in the nail changes [7]. The inclusion of peripheral enthesitis into classification criteria in 1990 and 1991 marked important milestones in the recognition and assessment of SpA [8,9].

A major conceptual advance occurred in 2001, when the “enthesis organ” (or “entheseal body”) model was proposed, expanding the definition beyond the insertion site itself to include adjacent structures, such as bursae, fat pads, fibrocartilage, and subchondral bone, that function collectively to dissipate mechanical stress [10]. This broader anatomical concept explains why entheseal inflammation is often accompanied by adjacent soft-tissue and bone changes, including bursitis, bone marrow edema, and cortical changes (erosions/enthesophytes).

Despite the pathogenetic relevance, entheseal involvement was long underrecognized because of the limited sensitivity of clinical examination. The advent of modern imaging has substantially modified this perspective. In particular, magnetic resonance imaging (MRI) and musculoskeletal ultrasound (MSUS), have demonstrated superiority over clinical assessment in detecting both inflammatory and structural abnormalities at entheseal sites [7]. More recently, advances in US technology, i.e., higher-resolution transducers, improved Doppler sensitivity, and refined image processing, together with a deeper understanding of anatomy and sonoanatomy, have dramatically broadened the concept of the enthesis. It is increasingly recognized that any transition from soft to hard tissue may display entheseal characteristics, and entheseal-like features have been described in anatomical sites beyond classical tendon insertions [11]. In this context, US is not only a sensitive tool for detecting peripheral enthesitis but also may offer the potential to move from a morphological assessment toward a more nuanced “sonohistological” evaluation, thereby expanding our understanding of entheseal biology in health and disease.

This narrative review aims to synthesize the most recent evidence on MSUS application to newly described entheseal sites in distal extremities and reassess current definitions of enthesitis, which remain largely centred on large peripheral insertions. Particular attention will be given to emerging US technologies, including advanced Doppler modalities, elastography, and molecular imaging. By addressing existing conceptual and diagnostic gaps, this review seeks to provide rheumatologists and sonographers with updated and clinically relevant evidence for comprehensive entheseal assessment.

## 2. Methods

The methodological approach of this structured narrative review followed the recommendations of the Scale for the Assessment of Narrative Review Articles (SANRA) [12]. A literature search was independently conducted by two rheumatologists (L.C. and C.R.) using PubMed database to identify studies addressing US imaging and emerging US-based techniques for the evaluation of the peripheral enthesis in distal extremities, last search December 2025. Discrepancies between reviewers were resolved by discussion and, when needed, a third reviewer (I.M.) was consulted. The search strategy combined controlled vocabulary and free-text terms related to the enthesis, anatomy of the distal extremities and US imaging, i.e., “enthesis”, “enthesis organ”, “enthesitis”, “enthesopathy”, “wrist”, “hand”, “fingers/thumb/toes”, “ankle”, “pulleys”, “retinacula”, “ligaments”, “tendons” and, US-related terms such as “ultrasound”, “ultrasonography”, “high-frequency” and “ultra-high-frequency ultrasound”, “sonoanatomy”, “anatomical landmarks”, “Doppler ultrasound”, “microvascular imaging”, “elastography (including strain and shear-wave elastography)”, “contrast-enhanced ultrasound”, “molecular ultrasound imaging”, and “ultrasound-guided procedure”, “ultrasound-guided biopsy”.

Eligible studies included English-language publications involving human subjects published within the previous 20 years. Original research articles, review papers, letters, and case reports or case series were considered to comprehensively capture new evidence and emerging techniques. Earlier seminal landmark studies were included when necessary to provide historical perspective and foundational knowledge. Conference abstracts and non-human studies were excluded in order to prioritize clinically relevant and higher-quality evidence.

Titles and abstracts were screened for relevance, duplicates were removed, and full texts were reviewed (Supplementary Material S1). The retrieved evidence was synthesized thematically to address the following areas: (i) US imaging (B- and Doppler mode) of emerging entheseal targets; (ii) US-guided procedures involving peripheral entheses; and (iii) advanced US-based techniques, including elastography and molecular imaging.

## 3. Results

**Figure 1:**
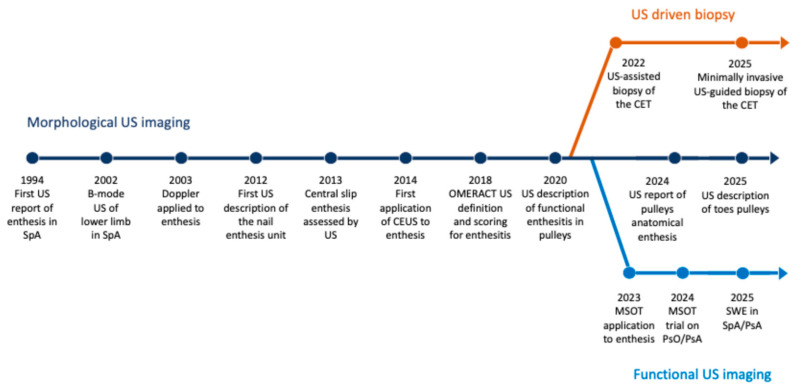
Timeline summarizing seminal papers in US assessment of the enthesis in rheumatology. Each timepoint in the timeline corresponds to a seminal publication listed in the references as follows: i. dark blue line [12,14,15,16,17,18,19,20,21,22]; ii. orange line [23,24]; and iii. light blue line [25,26,27,28]. CET: common extensor tendon; MSOT: multispectral optoacoustic tomography; PsA: psoriatic arthritis; PsO: psoriasis; SpA: spondyloarthritis; SWE: shear wave elastography; US: ultrasound.

Since the first report of US-detected entheseal involvement in patients with SpA in 1994 [13], the field has expanded substantially, with a steadily growing body of literature in rheumatology. In this narrative review, a total of 55 studies were analyzed and thematically synthesized into three main domains, Supplementary Material S2 summarizes all included studies. First, studies focusing on the characterization of entheseal sites using US and Doppler techniques, including increasing attention to newly described anatomical structures. Second, studies addressing invasive procedures targeting the enthesis in inflammatory diseases. Third, studies exploring ancillary approaches, including advanced US-based technologies and complementary imaging modalities, such as elastography and molecular imaging, which further enhance the diagnostic and investigative potential of US in entheseal assessment (Figure 1).

The assessment of the enthesis by US largely depends on the technical characteristics of the equipment, as these insertions are superficial structures that require high-frequency transducers for optimal visualization. In MSUS imaging, high-frequency US generally refers to probes operating above 14 MHz. Traditionally, most studies within the field of rheumatology, have focused on large and easily accessible entheses, such as the Achilles tendon, plantar fascia, quadriceps tendon, proximal patellar tendon, triceps tendon, and common extensor and flexor tendons insertions at the lateral and medial humeral epicondyles [29].

However, the introduction of very high- and ultra-high-frequency US systems, with transducers ranging from approximately 22 to 70 MHz, has reshaped the field of MSUS. While conventional probes up to 18–20 MHz allow visualization of structures located up to about 2 cm beneath the skin with submillimetric resolution, very high- and ultra-high-frequency technologies can achieve spatial resolution approaching 30 μm. This marked improvement has opened new opportunities for the detailed evaluation of superficial small anatomical structures [30].

Accordingly, recent studies have increasingly explored previously underrecognized entheseal sites in the distal extremities, particularly in the hands and feet. These regions are anatomically superficial, easily accessible to US imaging, and frequently involved in inflammatory conditions such as PsA. In particular, lesions occurring in the digits, including dactylitis, are thought to be driven by polyenthesitis, consisting in the inflammation of multiple small entheses within the same anatomical unit [14].

### 3.1. New Distal Entheseal Targets and Advanced Technologies for Assessing Entheseal Vascularization

**New entheseal targets in hands and wrists.** In 2013, central slip enthesitis (CSE) was described as a specific US lesion characterized by hypoechogenicity and thickening of the extensor tendon insertion at the base of the proximal phalanx on grey-scale imaging, variably associated with moderate-to-severe power Doppler (PD) signal at the enthesis and concomitant synovitis of the corresponding proximal interphalangeal joint. This lesion was reported in association with PsA [31]. Subsequent studies employing high-frequency US transducers (up to 18 MHz) further investigated this structure. Zabotti and colleagues demonstrated that CSE represents a discriminative lesion for distinguishing PsA from RA in the early stage of disease [32]. These observations were later reinforced by the identification of enthesitis of the distal extensor slip at the distal phalanx as another lesion highly specific for PsA [33]. These findings have contributed to the concept of “mini-entheses” of the hand, small anatomical structures that recapitulate the histological and US characteristics of larger entheses and now represent relevant targets for imaging in SpA.

Among the small entheses of the fingers, the pulley system, particularly the digital annular pulleys (DAP), has been increasingly investigated in recent years. These structures represent an interesting model from a biomechanical and anatomical perspective, as they function both as functional and anatomical entheses, with potential relevance for the study of the SpA group. Functional entheses are sites where the typical features of an enthesis organ, such as periosteal and sesamoid fibrocartilage, are present even in the absence of a direct tendon insertion into bone, whereas anatomical entheses correspond to true tendon insertions at the bone surface [15].

US has proven to be an effective imaging modality for visualizing these extremely small structures, particularly when high- and very high-frequency transducers exceeding 20 MHz are employed [16]. In anatomical validation studies conducted on cadaveric specimens, US measurements of pulley thickness showed a strong correlation with anatomical measurements obtained using a digital calliper, which represents the gold standard technique. These findings highlight the remarkable capability of US to assess submillimetric anatomical structures in vivo with high precision [34].

The potential involvement of DAP in PsA was first explored in 2018, when increased pulley thickness was observed in patients with established PsA compared with healthy controls and RA. In that study, US examination was performed using a 22 MHz transducer and focused on the A1, A2, and A4 pulleys, suggesting the presence of intrinsic structural changes that could contribute to disease pathogenesis. Further evidence supporting the inflammatory involvement of these structures emerged from studies reporting intra-pulley PD signal in patients with clinically active dactylitis [16]. In line with these observations, the pulley system has recently been incorporated into the Global OMERACT Ultrasound Dactylitis Score (GLOUDAS), reinforcing its relevance for imaging assessment in PsA [35].

The true entheseal nature of DAP insertions was formally characterized only in 2025. In that study, US was shown to be an optimal imaging tool to identify the insertional areas of pulleys not only in the fingers but also in the thumb, despite the latter presenting a more complex anatomical configuration. Notably, a landmark-based sonographic description of pulley entheses was proposed, relying on readily identifiable anatomical structures. Specifically, the distal palmar fold, volar plate, and sesamoid bones were used as landmarks for the A1 pulley enthesis; the bony ridges of the proximal phalanx and the exit of Camper’s chiasm were used to identify the A2 pulley enthesis; and the ridges of the middle phalanx together with the insertion of the flexor digitorum superficialis tendon were used to localize the A4 pulley enthesis. To overcome technical difficulties related to anisotropy when imaging these small insertions, particularly at the level of the A2 and A4 pulleys, a specific dynamic maneuver, the so-called contralateral rotation maneuver, was introduced. After obtaining optimal visualization of the pulley in the transverse plane, a composite movement combining rotation of the finger with a simultaneous sliding of the probe by 30–45° in the opposite direction was performed to ensure perpendicular insonation of the entheseal region [34]. The pulley system of the thumb has been investigated from a rheumatological perspective in only one study, which confirmed findings similar to those observed in triphalangeal fingers while emphasizing the greater technical difficulty of evaluating this region [36]. The clinical relevance of identifying pulley entheses was subsequently highlighted in a report describing the presence of PD signal within the anatomical enthesis of a digital pulley in a patient with early PsA, confirming that these structures may represent important inflammatory targets [37].

Beside tendons and pulleys insertions, fingers display a complex set of ligaments, which are involved in granting stability and restraining motion stress. The collateral ligaments of the metacarpophalangeal and interphalangeal joints are easily accessible to US and have fibrocartilaginous entheses [38]. Two main structures compose this complex: the proper collateral ligaments (PCLs) and the accessory collateral ligaments (ACLs)**.** The PCL originates from the metacarpal or interphalangeal head and inserts on the lateral base of the adjacent distal phalanx. The ACL runs from the metacarpal or interphalangeal head to the volar plate. Both ligaments are intimately related to the joint capsule [39]. In psoriatic dactylitis, high-resolution MRI studies revealed collateral ligament enthesitis as a marker of digit involvement in 75% of fingers [14]. In line with these results, MSUS detected finger collateral enthesitis, especially in late-onset SpA group (≥57 years at a median age) [40]. Moreover, US-detected enthesitis at the collateral ligaments is clinically relevant, correlates with physical findings, and is predictive of PsA diagnosis in psoriasis (PsO) patients with hand arthralgia [41]. 

Taken together these data suggest an important role of collateral ligaments in the pathogenesis of dactylitis. Consequently, their evaluation was integrated into the GLOUDAS scoring system [35]. 

Additional potential entheseal sites have been described at the wrist. In particular, the insertion of the flexor carpi ulnaris (FCU) at the pisiform represents a complex fibrocartilaginous enthesis, as anatomical studies have identified five fibrocartilaginous attachments in this region, the largest corresponding to the FCU tendon while the others belong to adjacent ligaments [42]. The US features of pathology at this site have been explored in a small radiological study using 13–16 MHz linear transducers with PD evaluation. Nine patients referred from a rheumatology clinic were examined, including five with inflammatory rheumatic diseases (RA and SpA) and four with painful FCU insertion related to mechanical overload. Peritendinous effusion, pisiform cortical irregularities suggestive of erosions, and peritendinous soft-tissue thickening at the FCU insertion were observed exclusively in patients with rheumatic disease [43]. However, the absence of a standardized definition of enthesitis and the very limited sample size prevent firm conclusions regarding the clinical relevance of the reported data. In addition, the definition of peritendinous effusion is questionable, as the FCU does not have a true synovial sheath, making the US findings described more probably a paratenonitis. 

Finally, the nail apparatus has been involved in the development of the SpA/PsA group, as the distal interphalangeal (DIP) joint represents a unique anatomical region where the nail unit, extensor tendon, and bone form an integrated functional structure, the “nail-enthesis unit” [44]. Histological studies showed that extensor tendon fibres extend beyond their insertion into the distal phalanx to envelop the nail root and matrix, forming a continuous connective tissue network [45]. This supports the hypothesis that nail disease in PsA may reflect inflammation arising from the adjacent enthesis rather than a purely cutaneous process. However, direct longitudinal evidence remains limited.

MSUS allows the non-invasive assessment of the nail complex. The nail plate is visualized by US as a trilaminar structure composed of two parallel hyperechoic lines (dorsal and ventral plates) separated by a thin hypoechoic space. The nail bed appears as a homogeneous hypoechoic layer beneath the ventral plate, while the nail matrix is located proximally and may be less distinctly delineated. Under normal conditions, PD signal is minimal or absent, although low-grade signals may occur depending on technical and patient-related factors [17].

US can detect subclinical nail abnormalities not evident on physical examination [46], and has been proposed as a tool to identify patients at risk of progression from PsO to PsA [47], as well as to detect subclinical enthesitis [48]. However, its predictive value remains uncertain due to heterogeneity in study design and methodology.

Compared with clinical indices, such as the Nail Psoriasis Severity Index (NAPSI), US provides a more comprehensive evaluation by quantifying nail bed and matrix thickness and assessing vascularity. This enables simultaneous analysis of structural and inflammatory changes. Standardized scoring systems, such as the Brown University Nail Enthesis Scale (BUNES), have been developed to integrate morphologic and Doppler findings [49], although they are not yet widely adopted and require further validation. 

Structural abnormalities are commonly described using the Wortsman classification [50], which includes focal hyperechoic changes, loss of ventral plate definition, wavy nail plates, and complete loss of definition. Nail bed vascularization is typically graded semi-quantitatively based on PD signal extent (<25%, 25–50%, and >50%) [51]. However, these findings are not entirely specific, as similar features may be observed in healthy individuals. 

Increased nail bed and matrix thickness, along with enhanced PD signal, are generally reported in PsO and PsA [48,52,53,54,55,56]. Morphological changes appear to have better discriminatory value, whereas PD findings are more variable and influenced by technical factors (i.e., machine sensitivity and settings), raising concerns about standardization. 

Technical aspects are critical for optimal imaging. Most studies use frequencies between 15 and 24 MHz, although higher frequencies improve resolution and visualization of nail structures. Despite this, access to ultra-high-frequency probes remains limited.

Nail US sensitivity to change has been explored in therapeutic studies. Systemic therapies such as acitretin and methotrexate improve ultrasound parameters, although acitretin is less effective on entheseal inflammation [57,58], while apremilast reduces structural abnormalities over longer follow-up [59].

More recently, Ruscitti et al. showed that ultra-high-frequency (27 MHz) US is effective for monitoring treatment response in early PsA, with significant improvements in inflammatory parameters, including BUNES PD score, after 24 weeks, while structural changes remained stable. Baseline ultrasound findings correlated with clinical disease activity, suggesting greater responsiveness of inflammatory features [18].

Overall, nail US is a promising tool for evaluating the nail-enthesis unit, but further standardization and longitudinal validation are required before routine clinical implementation.

**Figure 2:**
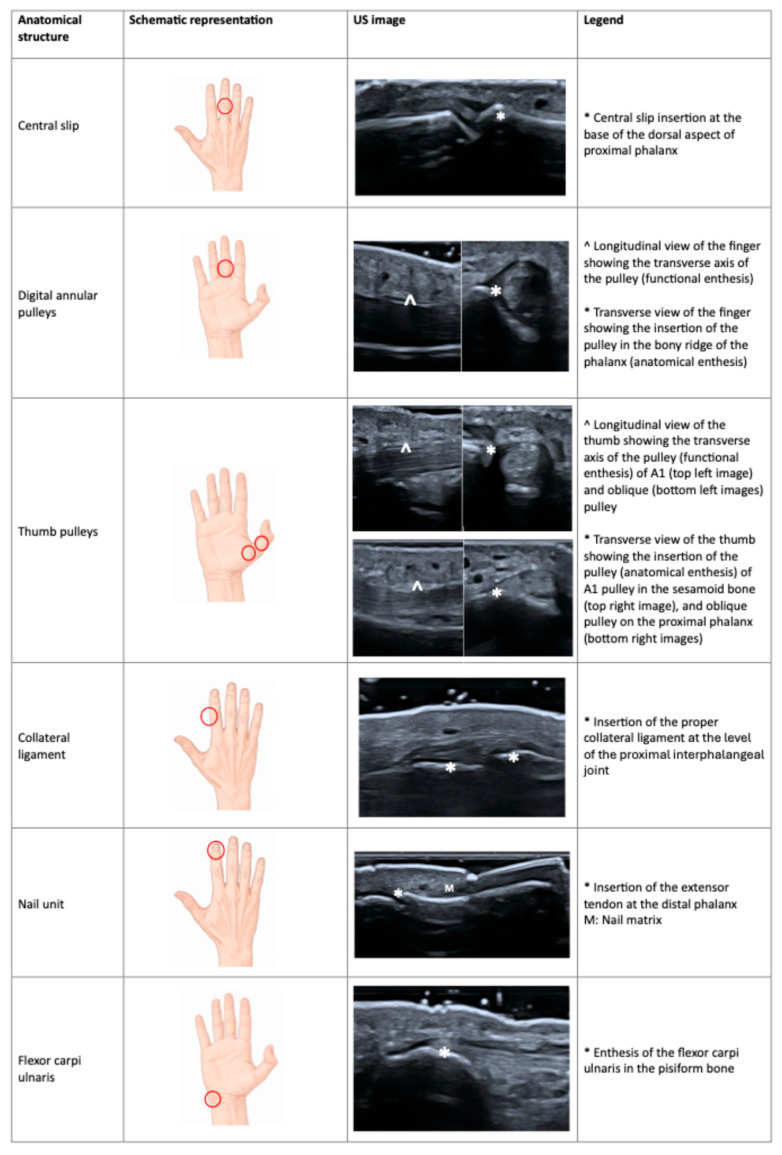
Schematic representation and US images of the new entheseal site described in hands and wrists. Red circles indicate the anatomical site to scan in order to assess the entheses. US: ultrasound.

Figure 2 highlights newly described entheseal structures in hands and wrists.

**New entheseal targets in foot and ankle.** Beyond the large entheses traditionally assessed at the ankle and foot, high-frequency US has recently enabled the exploration of smaller functional and anatomical entheses. Among these, the flexor tendon pulley system of the toes has emerged as a potential imaging target, similarly to what has previously been described in the hand. Anatomical dissections have shown a consistent pulley configuration in the toes, with four annular pulleys (A1–A4) and two cruciform pulleys (C1–C2), typically identified in the lesser toes, whereas the great toe usually presents three annular pulleys (A1–A3) together with two cruciform pulleys. High-frequency US performed with linear probes in the 18–24 MHz range has confirmed the anatomical location of these structures in vivo. Using this approach, the annular pulleys of the first to fourth toes can be consistently visualized, whereas identification in the fifth toe is less reliable due to its smaller size and the reduced distance between adjacent pulleys, which limits probe maneuverability. Beyond their anatomical characterization, preliminary clinical observations suggest that the pulley system may also be involved in inflammatory disease. In a case reported by the same authors, US examination of a patient with SpA revealed joint effusion and synovial hypertrophy at the ankle and metatarsophalangeal joints, associated with Doppler hypervascularization and diffuse soft-tissue edema. In addition, a markedly thickened A1 pulley (3.1 mm) was identified at the level of the great toe, which was also affected by nail dystrophy. These findings suggest that, as already described in the fingers, toe pulleys may represent additional small entheseal targets detectable with high-frequency US in inflammatory conditions affecting the foot [60].

Other peri-ankle structures have also been proposed as potential entheseal sites of interest. The tendons surrounding the ankle play an essential role in foot biomechanics and in the distribution of mechanical forces across the foot, and their bony insertions may represent additional sites involved in inflammatory processes. Nevertheless, MSUS assessment of these specific entheses remains limited in rheumatology. A study published in 2017 evaluated the entheses of the peroneus brevis tendon at the base of the fifth metatarsal and the tibialis posterior tendon at the navicular bone using a 15 MHz transducer. To optimize visualization of these insertions, the authors proposed a dedicated scanning technique in which the transducer was first positioned perpendicular to the skin in the longitudinal plane and then tilted approximately 45° cephalad while maintaining the same orientation. This maneuver helped reducing anisotropy and the apparent hypoechogenicity commonly encountered during standard imaging, improving visualization of the superficial tendon fibres and facilitating a more accurate assessment of these anatomically complex insertions. When applied to patients with SpA and RA, abnormalities were reported more frequently than in healthy controls. The tibialis posterior tendon enthesis was the most frequently affected site, with fibre disruption observed in more than 20% of patients, while PD signal was detected in approximately 15% of individuals with RA and 5% of those with SpA. However, the study did not provide a clear definition of enthesitis and did not explore correlations between US findings and clinical disease activity. In addition, the lack of stratification among different SpA subtypes prevents conclusions regarding potential differences between conditions such as PsA and axial SpA [61].

Finally, US evaluation of ankle retinacula has also been proposed as an extension of entheseal imaging in inflammatory arthritis. The main retinacular structures include the superior peroneal retinaculum laterally and the flexor retinaculum medially, both of which may function as enthesis-like structures because of their fibrous attachment to bone. Under physiological conditions, these structures are thin, typically measuring less than 1 mm. In a comparative study including patients with RA, PsA, and healthy controls, US examination was performed using a 12–18 MHz transducer to assess retinacular thickness, echogenicity, and vascularization. In the absence of a previously established definition, the authors introduced the term “retinaculitis,” defined by retinacular thickening greater than 1 mm associated with hypoechogenicity and/or PD signal. The study demonstrated that abnormalities predominantly involved the flexor retinaculum, which was significantly thicker in PsA compared with both RA and controls (0.96 ± 0.39 mm vs. 0.64 ± 0.15 mm and 0.56 ± 0.12 mm, respectively). Additional findings, including hypoechogenicity, Doppler signal, and periosteal bone proliferation at the retinacular insertion, were also more frequently observed in PsA [62]. In contrast, no relevant inflammatory changes were detected at the superior peroneal retinaculum. The preferential involvement of the flexor retinaculum may be related to the higher mechanical stress experienced by this structure, which stabilizes the posterior tibial tendon and prevents bowstringing, thereby contributing to maintenance of the medial longitudinal arch of the foot. Despite these observations, the evaluation of ankle retinacula remains largely unexplored. In addition to the flexor and superior peroneal retinacula, the ankle complex includes the superior and inferior extensor retinacula anteriorly and the inferior peroneal retinaculum laterally [19], which may represent additional potential targets for high-frequency MSUS investigation.

**Figure 3:**
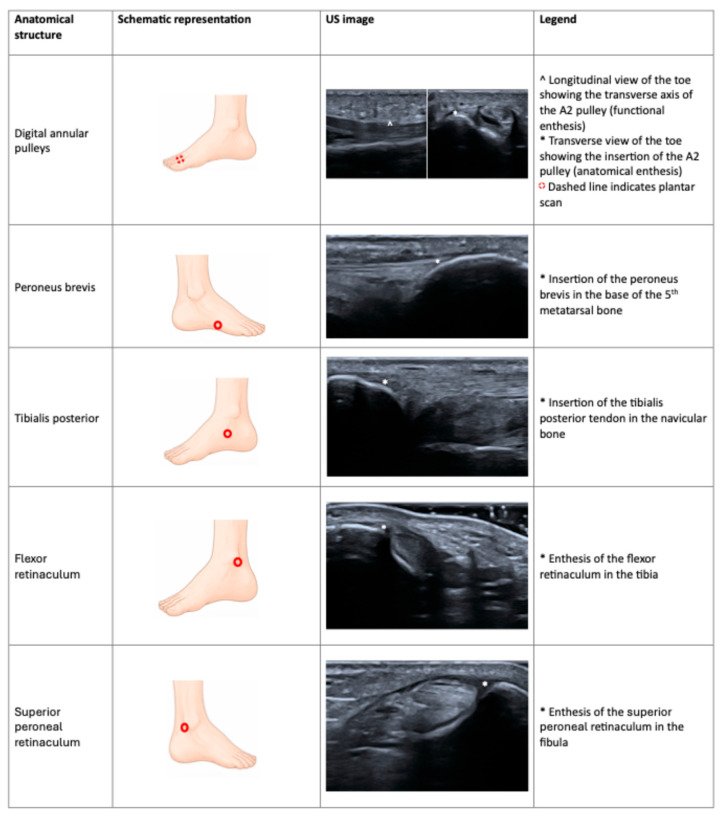
Schematic representation and US images of the new entheseal site described in feet and ankles. Red circles indicate the anatomical site to scan in order to assess the entheses. US: ultrasound.

Figure 3 summarizes recently identified entheses in ankles and feet.

**New US tools to assess entheseal vascularization.** Doppler techniques assess entheseal vascularization as a marker of active inflammation when the signal is located within 2 mm from the cortical bone, as defined by OMERACT [63]. In this context, emerging microvascular imaging technologies, i.e., Superb Microvascular Imaging (SMI), have shown increased sensitivity in detecting low-velocity and small-vessel blood flow compared with conventional Doppler modalities, without requiring contrast agents [64]. Although these techniques have demonstrated superior performance in visualizing neovascularization across a range of tendinopathies (i.e., lateral epicondylitis, patellar and Achilles tendinopathy), with correlations to symptom severity and potential utility in treatment monitoring, their application in enthesitis remains largely unexplored. Evidence is currently limited to a single study in lateral epicondylitis due to mechanical overload [65], while SpA-induced enthesitis has still not been evaluated. Authors found that neovascularization of CET origin was detected much better with SMI compared to colour Doppler (CD) and PD modalities, and the combination of SMI and B-mode US was found to have excellent diagnostic performance. 

Interestingly, no studies to date have specifically evaluated or compared SMI with conventional Doppler in enthesitis related to SpA. Therefore, despite promising technical advantages, further research is required to define the role of microvascular imaging in the assessment of enthesitis, particularly at the level of distal extremities.

Vascularization can even be assessed by using contrast agent, such as sulphur hexafluoride microbubbles. Contrast-enhanced US (CEUS) can enhance the visualization of altered tissue perfusion in case of pathological processes, as evidenced in cancer and in inflammatory conditions, such as synovitis, even in the subclinical stage [20]. Few data are available for the enthesis organ. In a small exploratory study, 14 patients with axial SpA underwent CEUS evaluation of the lateral epicondyle. The assessment was performed using a two-step CEUS approach: first, contrast tuned imaging technology (CEUS-CnTI) was applied to preserve microbubbles, followed by high-power Doppler (CEUS-PD) to induce their destruction. The study demonstrated for the first time that US contrast agents can enhance the detection of vascular signals at the enthesis, thereby improving sensitivity for identifying enthesitis. Moreover, the detected vascular signal was shown to be modulated by nonsteroidal anti-inflammatory drug therapy, indicating responsiveness to treatment. CEUS also proved valuable in clarifying equivocal findings on PDUS, confirming vascularization when the signal was uncertain and supporting the absence of enthesitis when no Doppler signal was present [66]. Overall, these findings have highlighted the potential utility of CEUS in assessing inflammatory activity at the enthesis level. However, important limitations included the small cohort of patient and the assessment of only one enthesis. Indeed, the short half-life of contrast microbubbles prevented the study of several entheseal sites and, parallelly, the requirement for low-frequency transducers to optimize microbubbles visualization made unfeasible the assessment of more superficial structures, that require high-frequency for optimal spatial resolution. Future technological advances, particularly the development of smaller microbubbles, may enhance the feasibility and clinical applicability of CEUS in rheumatology.

### 3.2. US-Guided Procedures on Peripheral Enthesis 

The enthesis has long been regarded as an anatomical site largely inaccessible to biopsy, and most insights into the entheseal microenvironment have historically derived from specimens obtained during spinal surgery. The advent of MSUS, however, has transformed the feasibility of minimally invasive tissue sampling in rheumatology. US-guided synovial biopsies are now routinely performed across different disease stages, including remission, demonstrating the safety and reproducibility of US-guided approaches [67]. In contrast, only a limited number of studies have explored US-assisted or US-guided biopsy of the enthesis.

The first report was provided by McGonagle and colleagues, who performed biopsies of the plantar fascia and patellar tendon entheses after identifying local inflammation by MRI. Under real-time US guidance, a 16 G Jamshidi needle was used to obtain tissue samples. Histological analysis revealed abnormal entheseal architecture in patients with SpA, characterized by increased vascularity and cellular infiltration compared with controls. Macrophages predominated within the fibrocartilage, whereas lymphocytes were scarce at the insertion site, supporting the concept of a primarily innate immune-driven process [23].

Subsequently, in 2022, a study described biopsy of the CET enthesis using Blakesley forceps, after US localization of the target area. In this setting, US played an assisting role, enabling accurate identification of the enthesis prior to a surgically performed sampling procedure [24]. More recently, in 2025, our group proposed a standardized, landmark-based, fully percutaneous US-guided technique for CET entheseal biopsy. This approach, grounded in a detailed anatomical and sonoanatomical knowledge, allows controlled real-time instrument positioning at the level of the extensor carpi radialis brevis insertion, the region most commonly involved in inflammatory pathology. Bony landmarks, including the anterior tubercle of the lateral epicondyle, were used to reliably identify adjacent stabilizing structures, such as the radial collateral ligament, thereby maximizing procedural safety. Anatomical validation confirmed accurate sampling of the intended entheseal compartment, and the technique proved safe, feasible and reproducible [68].

Both assisted and fully guided approaches successfully yielded informative tissue for histological analysis. Notably, the technique proposed by Pachowsky and colleagues was then applied in the EBIO study, in which patients with PsA and lateral epicondyle enthesitis underwent biopsy before and after treatment with an interleukin-17 inhibitor. Six months after therapy, PD signals were reduced compared to baseline, paralleled by a marked modulation of the tissue microenvironment toward a non-inflammatory profile [69].

Although preliminary and based on small cohorts, these in vivo studies highlight the pivotal role of US when supported by a rigorous anatomical understanding. Beyond ensuring feasibility and safety, fully US-guided entheseal biopsy offers a unique opportunity to establish criterion validity for US assessment of the enthesis, using histology as the reference standard. By directly correlating imaging findings with tissue-level changes, this approach may refine the interpretation of structural and vascular abnormalities and strengthen the biological basis of US-defined enthesitis.

The main characteristics and comparative features of studies describing peripheral entheseal biopsy are summarized in Table 1.

### 3.3. Functional US-Based Imaging for Peripheral Entheses

Elastography is an US-based technique that allows non-invasive evaluation of the mechanical properties of soft tissues, particularly tissue stiffness, which may reflect structural remodelling or inflammatory changes. Up to date, most studies have been conducted in radiology and sport medicine settings with a specific focus on tendon pathology rather than enthesitis related to rheumatic conditions [70].

In MSUS imaging, two complementary approaches are commonly used: strain elastography (also known as sonoelastography) and shear-wave elastography (SWE), both of which have been applied to the study of peripheral entheses.

Strain elastography measures tissue deformation in response to manual or transducer-applied compression, producing a colour-coded map that provides a qualitative or semi-quantitative assessment of relative stiffness. Applied to the Achilles tendon, this technique can differentiate pathological from healthy tissue, with affected entheses showing a significant colour shift from green to blue/light blue. These increased stiffness patterns correlate with B-mode findings of fibrillar disruption and fragmentation extending to the insertion site [25].

In contrast, SWE generates shear waves via acoustic radiation force impulses and quantifies stiffness based on their propagation speed, yielding objective measurements in metres per second (m/s) or kilopascals (kPa). In patients with PsO, SWE has detected entheseal changes (mean stiffness ~80–92 kPa) earlier than conventional B-mode US or PD, which often appear normal in subclinical stages of inflammatory disease [26]. One study evaluating SWE of the plantar fascia demonstrated a modest improvement in diagnostic performance for detecting plantar fasciitis in axial SpA [71]. 

Normative data for healthy adults identify the Achilles tendon as the stiffest lower limb enthesis (median SWV 3.04–3.17 m/s), although age is a critical confounder, showing a moderate negative correlation with stiffness across multiple sites [72]. 

From a technical perspective, strain elastography is highly operator-dependent, and even SWE can be influenced by subjective transducer pressure, potentially producing pre-compression artefacts that artificially increase stiffness. Entheses are anisotropic, so shear wave velocities are higher and more variable in the transverse plane compared to the longitudinal plane. Larger region of interest (ROI) may include surrounding subcutaneous tissue or bone, resulting in higher and less consistent stiffness measurements. In strain elastography, results are relative; a healthy tendon may appear softer if adjacent to hard bone, stressing the need for standardized analysis areas.

Biological and procedural factors further affect accuracy. Patient positioning is critical, as joint flexion alters entheseal tension, i.e., knee flexion (45–60°) stretches the extensor mechanism, increasing stiffness compared to an extended position [73]. Age decreases collagen density, leading to reduced shear wave velocity, while lateral dominance and mechanical load can increase stiffness on the dominant side. Body mass index (BMI) and physical activity also modulate entheseal morphology and mechanical responses, with high BMI or athletic activity potentially altering stiffness values [27].

Current research is additionally constrained by methodological limitations. Most studies are cross-sectional, limiting the ability to predict progression from subclinical enthesopathy to clinical psoriatic arthritis or tendon rupture. Sample sizes are often small, single-centre, and skewed toward younger, low-BMI populations, reducing generalizability. The absence of histopathological correlation prevents definitive linkage of stiffness changes to specific inflammatory or degenerative processes. Deep entheses are also difficult to assess compared with superficial sites such as the Achilles tendon.

To improve reproducibility and reduce pre-compression artefacts, recent methodological recommendations include performing SWE with the patient supine and the knee extended, using a 2.0 mm diameter ROI to avoid volume averaging from surrounding tissues, and acquiring images in the longitudinal plane with a thick layer of coupling gel, but further studies are needed to standardize the technique [73].

Overall, elastography enhances US by combining structural and functional assessment of the enthesis, offering unique insights into biomechanical and tissue alterations. Nevertheless, rigorous methodological research, longitudinal studies, and validation against histopathology are essential before elastography can be routinely integrated into clinical assessment of peripheral entheses to complement US assessment.

Among emerging imaging approaches, multispectral optoacoustic tomography (MSOT) is a hybrid modality that combines conventional US with optoacoustic imaging. By exposing tissues to near-infrared laser pulses at multiple wavelengths, this technique detects the optical absorption spectra of endogenous chromophores, i.e., oxyhaemoglobin, deoxyhaemoglobin, lipids, and collagen. The resulting signals enable quantitative assessment of tissue metabolic composition, while US simultaneously provides high-resolution anatomical localization. Through this integration, MSOT allows the detailed spatial characterization of the enthesis to be combined with in vivo functional and metabolic assessment, offering a novel perspective for investigating peripheral entheses. Preliminary applications of MSOT at entheseal sites have revealed distinct metabolic signatures, including increased hemoglobin content, higher oxygen saturation, and enhanced collagen signals under inflammatory conditions such as PsA. Indeed, sonographically detected enthesitis corresponded to MSOT findings suggestive of vascular remodelling and fibrocartilage alterations within the enthesis [28]. Further insights were provided by the MAPSA trial, which evaluated metabolic changes at several entheses, including the lateral humeral epicondyle, distal patellar tendon insertion, and Achilles tendon, in individuals with PsO, PsA, and healthy controls. Patients showed increased vascular signals and collagen turnover compared with controls, with more pronounced alterations in PsA. Notably, site-specific differences were observed, with the Achilles enthesis displaying the greatest metabolic divergence between patients and healthy subjects [74].

Despite these promising observations, several limitations remain. Acquisition protocols and outcome measures are not yet standardized, and image reconstruction algorithms require further optimization to reduce artefacts inherent to optoacoustic imaging. In addition, signal reliability may be influenced by skin pigmentation, and the high cost and limited availability of MSOT equipment currently limit clinical use. Consequently, further technical refinement and dedicated validation studies focusing specifically on entheseal assessment are required before this technique can be incorporated into entheseal imaging.

## 4. Discussion

The findings summarized in this review highlight a rapidly evolving landscape in the assessment of the enthesis. This evolution is particularly evident at the level of distal extremities, where previously underrecognized or undescribed entheses are now being explored. The increasing availability of high- and very high-frequency US transducers has been instrumental in enabling the visualization of millimetric and submillimetric structures, thereby expanding the anatomical and pathological spectrum accessible to US [75]. In parallel, the integration of advanced Doppler techniques and emerging microvascular imaging methods is challenging traditional definitions of enthesitis, especially in very small anatomical sites.

A key issue concerns the assessment of active inflammation. By current definition, Doppler signal is considered indicative of enthesitis when detected within 2 mm from the bony cortex [63]; however, this criterion is difficult to apply to very small entheses, such as pulley systems or collateral ligament insertions. In these contexts, grey-scale abnormalities, such as thickening and hypoechogenicity, may be more readily and reliably assessed. At the same time, the introduction of highly sensitive microvascular imaging techniques necessitates careful differentiation between physiological and pathological vascularization, as well as the establishment of standardized thresholds to avoid overestimation of inflammatory activity [76]. So, vascularization stands out as a major point to consider in future research on enthesis inflammation. Despite the growing number of newly described entheses, the available evidence remains limited. Most studies are based on small cohorts, often represent single-centre experiences, and frequently lack methodological robustness. Anatomical validation, including cadaveric correlation and histological confirmation, is rarely performed, and comparisons with established imaging modalities are largely absent. These limitations highlight the need for rigorous validation studies. Standardization represents a major unmet need in this field. Consensus on image acquisition protocols, definition of normal and pathological findings, and documentation of disease-related changes is essential [21]. Importantly, newly identified entheses may improve diagnostic accuracy and disease characterization in rheumatology. This will likely require revisiting and adapting the definition of enthesitis according to anatomical site. Similarly, current US scoring systems for enthesitis may require updating. Early tools such as the Glasgow Enthesitis Scoring System (GUESS) focused exclusively on grey-scale changes and demonstrated limited sensitivity to change [22]. Subsequent indices, including the D’Agostino score and the Madrid Sonographic Enthesitis Index (MASEI), incorporated Doppler findings and broader structural assessment [77,78], while the Spanish Enthesitis Index adopted a patient-level approach [79]. However, none of these systems includes the newly described small entheses of distal extremities. The GLOUDAS score represents a partial exception, incorporating functional entheses, such as pulleys and collateral ligaments, in the assessment of dactylitis in PsA, although without direct evaluation of pulleys insertions [35]. Recently, the importance of US assessment across different entheseal sites has further stressed that both lesion type and the topographic distribution of involvement contribute to improving discrimination between SpA and healthy individuals, including subjects with age or mechanically related entheseal changes. Particular attention has been drawn to key sites such as the distal patellar tendon insertion, the Achilles tendon enthesis, and common extensor tendon insertions, which appear to provide high discriminatory value and support a site-specific approach to enthesis evaluation. This lesion-oriented and topographic perspective also suggests that assessment of both conventional and newly described entheses should inform future refinement of US scoring systems [80]. Overall, there is still no consensus on the optimal scoring system, and existing tools may not fully capture the complexity of entheseal involvement.

The pursuit of deeper biological insight has also led to the development of minimally invasive, US-guided biopsy techniques targeting the enthesis. Although still at a preliminary stage, these approaches may offer a unique opportunity to investigate the immunopathological mechanisms underlying enthesitis. Current data are limited to small series, lack comparison with healthy tissue, and are largely restricted to accessible sites, such as the lateral epicondyle. Nevertheless, these techniques may, in the future, contribute to patient stratification and personalized therapeutic approaches, analogous to advances seen in synovial tissue research [81].

Moreover, emerging adjunctive imaging modalities, such as MSOT, provide additional functional information by enabling the assessment of tissue composition and vascular status. While promising, these technologies require further validation, standardization, and broader accessibility before they can be integrated into routine clinical practice [82].

Finally, key strengths and limitations of this review should be acknowledged. Among its strengths, this structured narrative review provides an updated overview of an emerging field, integrating novel distal entheseal targets with advanced US applications and interventional approaches. However, limitations should also be recognized. As a narrative review, this work does not provide formal systematic synthesis or risk-of-bias assessment. The literature search was limited to PubMed, which may have resulted in the exclusion of studies indexed in other databases and introduced potential selection bias. However, PubMed was chosen for its extensive coverage of biomedical and rheumatology literature, and this limitation was mitigated through supplementary reference screening and expert input. Finally, much of the available evidence is heterogeneous and based on relatively small or exploratory studies, limiting the strength of some conclusions and reinforcing the need for further validation studies.

## 5. Conclusions and Future Perspectives

Research on the enthesis is evolving through the identification of novel anatomical sites and the application of advanced US technologies. Future research should focus on developing a comprehensive atlas of clinically relevant entheses, particularly in distal extremities, together with refining definitions and scoring systems for enthesitis. A gradual move from purely morphological assessment toward combined morphological and functional imaging may further expand evaluation by integrating vascular and potentially metabolic information.

US remains a uniquely versatile tool for the multilevel assessment of the enthesis. The use of high- and very high-frequency transducers has opened new perspectives for the evaluation of superficial and small structures, expanding both research opportunities and clinical applications. Continued technological innovation, coupled with methodological standardization and validation, will be essential to fully realize the potential of US in the assessment of enthesitis and related disorders.

## Figures and Tables

**Table 1:** Main characteristics of the published studies on US-guided/assisted entheseal biopsy.

	Study Design	Type of Procedure	Site of Biopsy	Ultrasound Role	Anatomical Validation	Histological Validation	Patients’ Inclusion	Disease	Feasibility Assessment	Safety Assessment	Complication
McGonagle D. et al., 2001 [23]	Proof-of-concept exploratory case series	Minimally invasive with a Jamshidi needle	Patellar tendon Plantar fascia	US guided—not detailed	No	Yes	5	SpA	Not assessed	Yes—one year follow up	No
Pachowsky M. et al., 2022 [24]	Prospective translational feasibility study (cadaveric validation + in vivo cohort)	Minimally invasive with a Blakesley forceps and blade incision	Common extensor tendon at the elbow lateral epicondyle	US assisted—to locate the CET enthesis	Yes	Yes	10	PsA	Yes	Yes—14 days follow up	Mild haematoma in 2 cases
Rizzo C. et al., 2025 [68]	Methodological standardization study (cadaveric cohort)	Minimally invasive with a nanoforceps and fully percutaneous	Common extensor tendon at the elbow lateral epicondyle	US-guided—fully detailed	Yes	Yes	No	NA	Yes	Yes	NA
Raimondo M.G. et al., 2026 [69]	Prospective, open-label, single-arm interventional study	Minimally invasive with a Blakesley forceps and blade incision Performed twice (before and after treatment, week 0 and week 24)	Common extensor tendon at the elbow lateral epicondyle	US assisted—to locate the CET enthesis	Yes	Yes	10	PsA	Yes	Yes—28 weeks follow up	No

CET: common extensor tendon, NA: Not applicable; PsA: psoriatic arthritis, SpA: spondyloarthritis, US: ultrasound.

## Data Availability

No new data were created or analyzed in this study.

## Supplementary Materials

The following supporting information can be downloaded at: https://www.mdpi.com/article/10.3390/jcm15103753/s1, Supplementary Material S1. Synthetic description of literature identification and selection. Supplementary Material S2. Main characteristics of the studies included.
