# Supplementary material for: From Invisible to Visible: Cutting-Edge Ultrasound Insights into Entheses of the Distal Extremities in Rheumatology
Source: J Clin Med. 2026 May 13;15(10):3753. doi: 10.3390/jcm15103753 (PMC13207774; doi:10.3390/jcm15103753)
Supplement: Supplementary file 1 [file jcm-15-03753-s001.zip › 01 Supplementary material S1 Enthesis .pdf]

Supplementary Material S1. Synthetic description of literature identification and selection

Identification

Records identified through PubMed search:	n = 184
Additional records (references/expert input):	n = 12
Total records identified:	n = 196

Screening

Duplicates removed:	n = 18
Records screened (title/abstract):	n = 178
Records excluded after screening (not enthesis/enthesitis-focused, not distal extremities, not US-based, conference abstracts, outside review scope):	n = 96

Eligibility

Full-text articles assessed:	n = 82
Full-text articles excluded (limited relevance to themes, not emerging techniques/targets):	n = 27

Inclusion

Studies included in narrative synthesis:	n = 55
