# Supplementary material for: From Invisible to Visible: Cutting-Edge Ultrasound Insights into Entheses of the Distal Extremities in Rheumatology
Source: J Clin Med. 2026 May 13;15(10):3753. doi: 10.3390/jcm15103753 (PMC13207774; doi:10.3390/jcm15103753)
Supplement: Supplementary file 1 [file jcm-15-03753-s001.zip › 01 Supplementary material S2 Enthesis 02052026.pdf]

Supplementary Material S2. Main characteristics of the studies included.

Study	Type of study	Anatomical site	Enthesis assessed	Modality	Population	Main findings	Main limitations
Filippou G et al, 2013 [31]	Letter/case report	Hand	Central slip	US	PsA	First description of central slip lesion	Case-based, exploratory
Zabotti A et al, 2016 [32]	Original	Hand	Central slip	US	Early PsA vs early RA	Discriminative value of the central slip lesion in early PsA	Single centre
Supundzhieva Y et al, 2025 [33]	Original	Hand	Central slip and distal slip	US	PsA, RA and HC	Mini-enthesitis of the central slip and distal slip is specific for PsA	Sample size, single centre
Tinazzi I et al, 2018 [16]	Original	Hand	Finger pulleys	US	PsA, RA and HC	Pulley thickening and PD signal are specific for PsA	Sample size, study limited to pulleys' functional enthesis
Coronel L et al, 2024 [34]	Original	Hand	Finger pulleys	US	Cadaveric and HC	US-anatomy-histology correlation and first description of pulleys' anatomical enthesis	Sample size, no patients included
Naredo E et al, 2024 [35]	Original	Hand	Digital entheses	US	PsA, cadaveric	Validated scoring system	Needs external validation
Coronel L et al, 2025 [36]	Original	Hand	Thumb pulleys	US	Cadaveric and HC	First characterization of thumb pulleys' anatomical entheses	Sample size, no patients included
Coronel L et al, 2025 [37]	Case report	Hand	Finger pulley	US	Early PsA	Finger pulley enthesitis at the anatomical enthesis identified as potential early sign of PsA	Single case
Benjamin M et al, 1998 [38]	Review	Enthesis	Fibrous and fibrocartilaginous enthesis	Anatomy, Histology	NA	Comprehensive overview of the entheseal organ (seminal paper)	No US description
Bouredoucen H et al, 2026 [39]	Review	Hand	Collateral ligament	US	NA	High frequency US allows the dynamic study of collateral ligaments at the metacarpophalangeal joint	No lesions' description
Endo Y et al, 2019 [40]	Original	Hand	Collateral ligament	US	SpA	Collateral enthesitis detected more frequently in late-onset SpA	Retrospective
Olivas-Vergara O, 2025 [41]	Original	Hand	Digital entheses	US	PsO patients with hand arthralgia	Digital mini-enthesitis is predictive of PsA development in PsO patients with hand arthralgia	Sample size, single centre
Adamczyk C et al, 2008 [42]	Original	Hand	Pisiform bone	Immunohistochemistry	Cadavers	Comprehensive histological characterization of the flexor carpi ulnaris enthesis at the pisiform bone (seminal paper)	No US description

Wick MC et al, 2011 [43]	Original	Wrist	Flexor carpi ulnaris	US	Patients with painful pisiform FCU insertion	Enthesitis at the flexor carpi ulnaris are specific of rheumatic diseases	Very small sample size, questionable definition of peritendinous effusion
McGonagle D et al, 2009 [44]	Review	Hand	Nail complex	MRI, Histology	NA	Description of anatomy, histology and fundamental concepts of the nail entheseal complex (seminal paper)	No US description
Tan AL et al, 2007 [45]	Original	Hand	Nail complex	MRI, Histology	PsA, Osteoarthritis and HC	The distal interphalangeal joint capsule is structurally integrated with the nail complex via enthesis-derived fibres, with MRI showing the dorsal capsular enthesis as the main site of inflammation (seminal paper)	No US description, limited sample
Wortsman X 2023 [17]	Review	Hand	Nail complex	US	NA	Overview of US applications to study the nail	Few data on PsA
Aydin SZ et al, 2012 [46]	Original	Hand	Nail complex	US	PsA, PsO and HC	Linked nail abnormalities and enthesopathy of the extensor tendon insertion at the distal interphalangeal joint	Sample size, single centre
Esposito M et al, 2025 [47]	Original	Hand	Nail complex	US	PsA, PsO	US of the nail may help in identifying PsO patients at risk of PsA	Single centre, cross-sectional, sample size
Ruscitti P et al, 2022 [48]	Original	Hand	Nail complex	US	PsA, PsO	US allows the detection of subclinical nail involvement in PsA patients without PsO and of subclinical entheseal alterations in PsO patients	Single centre, cross-sectional, sample size
Cunha JS et al, 2017 [49]	Original	Hand	Nail complex	US	PsA, PsO	Scoring system proposal for nail involvement	Needs validation
De Rossi SD et al, 2021 [50]	Original	Hand	Nail complex	US	PsA, PsO and HC	US detects structural abnormalities in patients with PsA and PsO	Cross-sectional, single centre
Gutierrez M et al, 2012 [51]	Original	Hand	Nail complex	US	PsA	Proposal of a power Doppler US composite score for the assessment of PsA	Needs validation, sample size
Acosta-Felquer ML et al, 2017 [52]	Original	Hand	Nail complex	US	PsA, PsO	Nail disease was associated with distal interphalangeal joint US enthesopathy	Single centre, sample size, cross-sectional
Idolazzi L et al, 2018 [53]	Original	Hand	Nail complex	US	PsA, PsO and HC	US detects nail abnormalities in PsA and PsO patients	Single centre, cross-sectional

Krajewska-Włodarczyk M et al, 2018 [54]	Original	Hand	Nail complex	US	PsA, PsO	US of the nail may help in identifying PsO patients at risk of PsA	Cross-sectional, sample size
Mondal S et al, 2018 [55]	Original	Hand	Nail complex	US	PsA and HC	US may detect subclinical nail abnormalities in PsA, with increased nail bed/matrix thickness correlating with nail disease severity	Single centre, sample size
Acer Kasman S et al, 2021 [56]	Original	Hand	Nail complex	US	PsA and HC	US is a feasible, reliable, and discriminative method to predict PsA	Needs external validation, sample size
Krajewska-Włodarczyk M et al, 2021 [57]	Original	Hand	Nail complex	US	PsO and HC	US may be useful to assess response to retinoid in nail PsO	Single centre, sample size
Krajewska-Włodarczyk M et al, 2018 [58]	Original	Hand	Nail complex	US	PsA, PsO	US of nail examination in PsA and PsO may help evaluate response to methotrexate	Sample size
Muñoz-Santos C et al, 2021 [59]	Original	Hand	Nail complex	US	Nail PsO	US detects nail improvement in patients with apremilast	Sample size, lack of control group
Ruscitti P et al, 2025 [18]	Original	Hand	Nail complex	US	Early PsA	US may provide additional information about the effectiveness of IL-17 and IL-23 inhibitors in psoriatic nail	Sample size, single centre, proof-of-concept
Pistoia F et al, 2025 [60]	Original	Foot	Toes' pulleys	US	Cadavers, HC and mixed patients	Comprehensive description of the sonoanatomy of the annular pulleys in the toes	Sample size, only three pathological cases
Ward IM et al, 2017 [61]	Original	Ankle	Tibialis posterior /peroneus brevis	US	RA, SpA and controls	Definition of optimal imaging technique and sonoanatomy of the tibialis posterior and peroneus brevis enthesis	Sample size, no clinical information
Forien M et al, 2024 [62]	Original	Ankle	Ankle retinacula	US	PsA, RA and HC	US retinacula abnormalities, appears specific of PsA	Sample size, not all retinacula
Bianchi S et al, 2019 [19]	Review	Ankle	Retinacula	US	NA	Comprehensive description of the ankle retinacula complex (seminal paper)	Pictorial essay, rheumatic patients not included
Balint PV et al, 2018 [63]	Original	Enthesis	Lateral condyle of the elbow, superior and inferior poles of the patella and calcaneal	US	PsA, SpA	Accepted inflammatory and structural components for US enthesitis definition (seminal paper)	Validation for large entheses

			insertion of the Achilles tendon				
Seskute G et al, 2023 [64]	Systematic review	Enthesis	Peripheral entheses	SMI	Rheumatic diseases	Summarizes emerging evidence on new Doppler technologies (seminal paper)	Heterogeneity of the included studies, no assessment of sensitivity to change
Arslan S et al, 2018 [65]	Original	Elbow	Common extensor tendon entheses	US, Doppler and SMI	Lateral epicondylosis	The combination of SMI and B mode US performs well in the assessment of lateral epicondylosis	Sample size, no rheumatic disease assessed
Polido-Pereira J et al, 2024 [20]	Original	Hand, wrist, elbow, knee, ankle, foot	Peripheral joints	CEUS	RA and HC	CEUS may be useful to detect subclinical disease in patients in remission (seminal paper)	Needs for normality cut-offs, not enthesis-specific
Mouterde G et al, 2014 [66]	Original	Elbow	Lateral epicondyle	CEUS	axSpA	CEUS improves the detection of inflammatory enthesitis in SpA patients with moderate disease activity	Sample size, no control group, only one enthesis assessed
Coletto LA et al, 2024 [67]	Original	Joint	Knee, wrist	US-guided synovial biopsy	RA	Description of a validated training protocol to perform synovial biopsies	Not enthesis-specific
McGonagle D et al, 2002 [23]	Original	Enthesitis	Plantar and patellar	US-guided entheseal biopsy	axSpA, uSpA, reactive arthritis	First description of entheseal biopsy	Sample size, poor description of the US assisted procedure
Pachowsky ML et al, 2022 [24]	Original	Elbow	Lateral epicondyle	US-assisted entheseal biopsy	Cadavers and PsA	Standardization of an US assisted entheseal biopsy	Sample size
Rizzo C et al, 2025 [68]	Original	Elbow	Lateral epicondyle	US-guided synovial biopsy	Cadavers	First description of a standardised, fully control US guided biopsy of the CET entheses	No patients included
Raimondo MG et al, 2026 [69]	Original	Elbow	Lateral epicondyle	US-assisted entheseal biopsy	PsA	First evaluation of enthesis response to treatment by repeated entheseal biopsy	Sample size
Albano D et al, 2024 [70]	Review	Tendons	Achilles, patellar, supraspinatus, long head of biceps, lateral epicondyle, extensor pollicis brevis, abductor pollicis longus	Elastography	NA	Elastography may increase sensitivity and diagnostic accuracy of conventional US imaging promoting early detection of tendinopathy	Only English records, no grey literature included, high heterogeneity of included studies
Sconfienza LM et al, 2010 [25]	Original	Tendon	Achilles tendon	Elastography, US	Painful Achilles tendon in athletes	Symptomatic tendons appeared stiffer and reduced elasticity was associated	Sample size, low-frequency transducer, risk of operator-

						with tendon fragmentation and loss of fibrillar structure<br>No differences between symptomatic and control tendons at the enthesis or the myotendinous junction	associated variations, absence of standardization
Temel E et al, 2025 [26]	Original	Tendon	Achilles tendon	Elastography, US	PsO and HC	Achilles tendons of PsO are stiffer than HC.<br>SWE may be effective in detecting subclinical enthesopathy and predicting the development of PsA in patients with PsO	Single centre, sample size, not clinical follow-up, risk of observer bias (single operator)
Daskareh M et al, 2025 [71]	Original	Foot	Plantar fascia	Elastography, US	axSpA and non-axSpA with and without plantar fasciitis (HC)	SWE may enhance the early identification of biomechanical changes in the plantar fascia	Sample size, cross-sectional
Mahazer MKJ et al, 2025 [72]	Original	Enthesis	Quadriceps, suprapatellar, infrapatellar, and Achilles entheses	Elastography	HC	SWE is a reproducible technique for quantifying enthesis stiffness. Age significantly influences shear wave velocity across different sites, with the Achilles enthesis consistently showing the highest values among all age groups	Single centre, sample size, single evaluation, study population skewed toward young individuals with low BMI
Leong SS et al, 2024 [73]	Original	Enthesis	Quadriceps enthesis	Elastography	HC	Establishment of a scanning protocol: supine position with extended knee extended; 2 mm diameter region of interest; image acquisition at the longitudinal plane	Sample size, no comparison with other imaging modalities, narrow demographic, only one entheseal site assessed
Römer C et al, 2023 [27]	Original	Tendon	Achilles tendon	Elastography	Healthy athletes	Achilles tendon stiffness is influenced by gender and by the type of sport	Sample size, no disease assessed
Tascilar K et al, 2023 [28]	Original	Enthesis	CET, patellar, quadriceps and Achilles tendon entheses	MSOT, US	PsA, RA and HC	MSOT detects biochemical differences in entheses showing clinical and sonographic	Lack of standardization, needs validation
Fagni F et al, 2024 [74]	Original	Enthesis	CET, quadriceps, patellar and Achilles tendon entheses	MSOT, US	PsA, PsO and HC	PsA and PsO patients present analogous metabolic patterns at the entheses that is exacerbated in the presence of inflammation	Only patients in low disease activity were assessed, needs standardization

Acronyms: axSpA: axial spondyloarthritis; BMI: body mass index; CEUS: contrast-enhanced ultrasound; CET: common extensor tendon; HC: healthy controls; IL: interleukin; MRI: magnetic resonance imaging; MSOT: multispectral optoacoustic tomography; NA: not applicable; PD: power Doppler; PsA: psoriatic arthritis; PsO: psoriasis; RA: rheumatoid arthritis; SMI: superb microvascular imaging; SpA: spondyloarthritis; SWE: shear wave elastography; uSpA: undifferentiated spondyloarthritis; US: ultrasound.
